# Abandonment and rapid infilling of a tide-dominated distributary channel at 0.7 ka in the Mekong River Delta

**DOI:** 10.1038/s41598-021-90268-6

**Published:** 2021-05-26

**Authors:** Marcello Gugliotta, Yoshiki Saito, Thi Kim Oanh Ta, Van Lap Nguyen, Toru Tamura, Zhanghua Wang, Andrew D. La Croix, Rei Nakashima

**Affiliations:** 1 Faculty of Geosciences, University of Bremen, 28359 Bremen, Germany; 2 MARUM, University of Bremen, 28359 Bremen, Germany; 3 Estuary Research Center, Shimane University, Matsue, 690-8504 Japan; 4 Geological Survey of Japan, AIST, Tsukuba, 305-8567 Japan; 5 HCMC Institute of Resources Geography, VAST, Ho Chi Minh City, Vietnam; 6 Graduate School of Frontier Sciences, University of Tokyo, Kashiwa, 277-8561 Japan; 7 State Key Laboratory of Estuarine and Coastal Research, ECNU, Shanghai, 200062 China; 8 Southern Marine Science and Engineering Guangdong Laboratory, Zhuhai, 519080 China; 9 Earth Sciences, School of Science, University of Waikato, Hamilton, 3240 New Zealand

**Keywords:** Sedimentology, Geomorphology, Climate sciences, Environmental sciences, Environmental social sciences, Natural hazards

## Abstract

The Ba Lai distributary channel of the Mekong River Delta was abandoned and infilled with sediment during the Late Holocene, providing a unique opportunity to investigate the sediment fill, timing and mechanisms of channel abandonment in tide-dominated deltaic systems. Based on analysis and age dating of four sediment cores, we show that the channel was active since 2.6 ka and was abandoned at 0.7 ka as marked by the abrupt disappearance of the sand fraction and increase in organic matter and sediment accumulation rate. We estimate that the channel might have been filled in a time range of 45–263 years after detachment from the deltaic network, with sediment accumulation rates of centimetres to decimetres per year, rapidly storing approximately 600 Mt of organic-rich mud. We suggest that the channel was abandoned due to a sediment buildup favoured by an increase in regional sediment supply to the delta. This study highlights that mechanisms for abandonment and infilling of tide-dominated deltaic channels do not entirely fit widely used models developed for fluvial-dominated environments. Their abandonment might be driven by autogenic factors related to the river-tidal and deltaic dynamics and favoured by allogenic factors (e.g., human impact and/or climate change).

## Introduction

The mechanism of channel abandonment in paralic environments is often explained by meander cutoffs to form oxbow lakes or by avulsions, largely based on observations from fluvial environments^1–5^. Tidal deltaic systems are often assumed to follow these models [e.g.,^6,7^]. Nonetheless, meander cutoffs and avulsions are rarer in large-scale tide-dominated deltas due to the relatively long-term stability and straight morphology of their distributary channels^8^, although meander cutoffs are relatively more frequent in the landward part of smaller-scale tide-dominated systems^9,10^. Due to this general misconception, mechanisms of channel abandonment in tide-dominated systems remain poorly understood. Moreover, little is known about the timing of infilling of abandoned channels as age data from these deposits are rare.

The Ba Lai is the only abandoned channel of the Mekong River Delta (MRD), which represents a unique case of a tide-dominated distributary channel that was abandoned in recent geological times. The Ba Lai has been almost entirely infilled with sediments while only a relict part of the channel still hosts water and is connected to the deltaic network through a small artificial canal. The palaeochannel margins are visible in satellite images due to the contrast in elevation and vegetation with the surrounding area and because of the discontinuity of beach ridges. The Ba Lai palaeochannel provides an excellent opportunity to investigate the sediment fill, timing and mechanisms of channel abandonment in tide-dominated deltaic systems, also in light of the contributing autogenic and allogenic factors. The present study, based on four sediment cores from the Ba Lai palaeochannel (Supplementary Information 1), will help to shed light on this topic.

## The Mekong River Delta (MRD)

**Figure 1:**
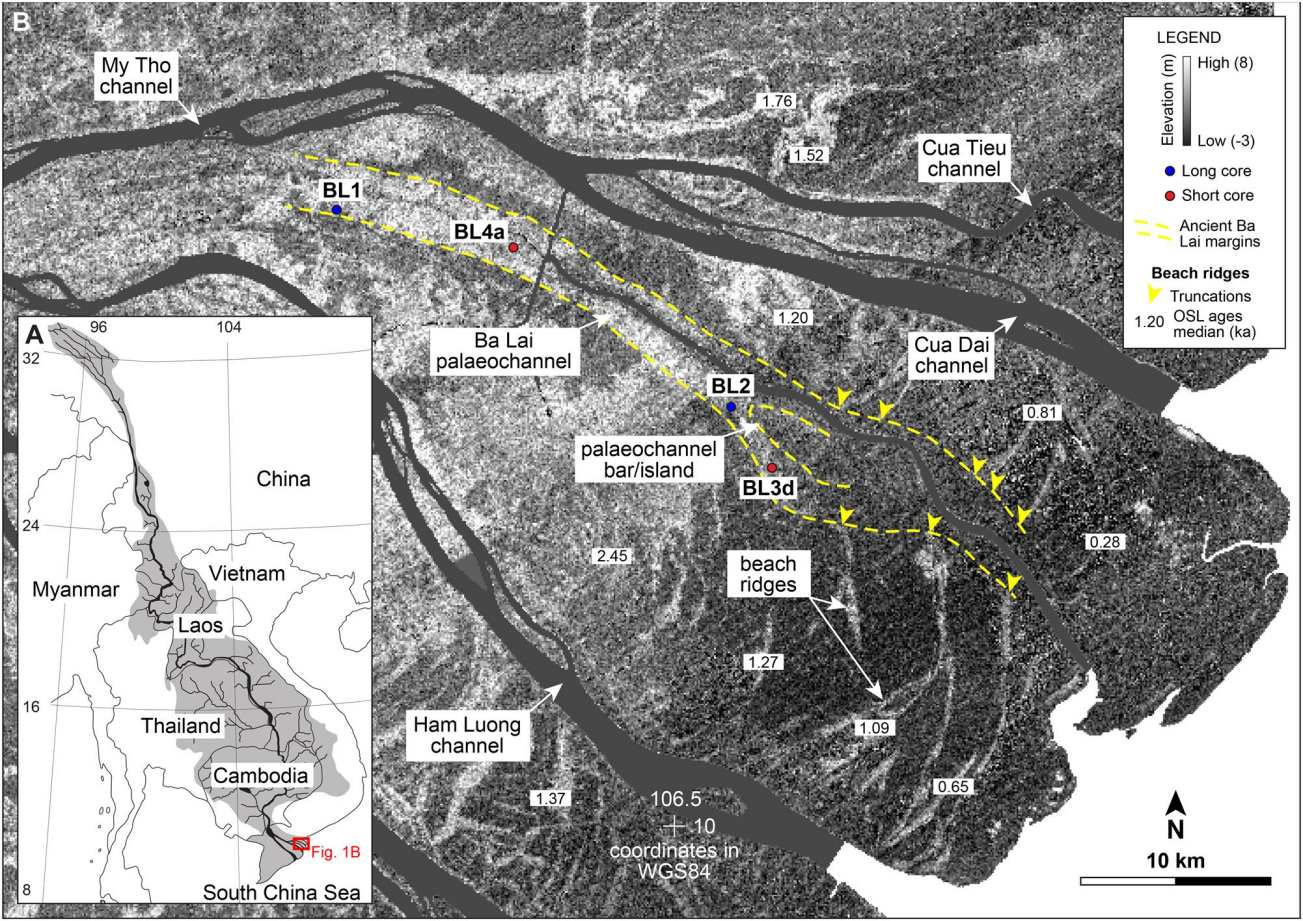
(**A**) Map of Southeast Asia with indication of the study area in southern Vietnam. (**B**) DEM from the SRTM of the north-eastern part of the MRD showing the margins of the Ba Lai palaeochannel and the locations of the four sediment cores from this study. Beach-ridge OSL ages are from^21^.

The MRD, in southern Vietnam (Fig. 1A), formed at approximately 8 ka in response to a deceleration in the global sea-level rise and, since then, it has prograded more than 200 km^11,12^. The present-day delta shows one of the world’s largest delta plains, which is intersected by numerous beach ridges, seven active distributary channels and the abandoned Ba Lai channel (Fig. 1B). The delta is a mixed-energy system characterised by an upstream to downstream transition from river-dominated to tide-dominated distributary channels^13,14^ and a wave- and tide-dominated shoreline^15,16^. The delta area and the lower drainage basin are characterised by a tropical savannah to monsoon climate of the Köppen-Geiger classification^17^, with a rainy summer generating high river discharge and a dry winter associated with low river discharge. Wind and wave direction and energy vary seasonally, with the summer monsoon generating weaker longshore currents toward the northeast and the winter monsoon displaying stronger longshore currents and an overall prevailing net sediment transport toward the southwest^16,18^.

## Sedimentary facies and ages

**Figure 2:**
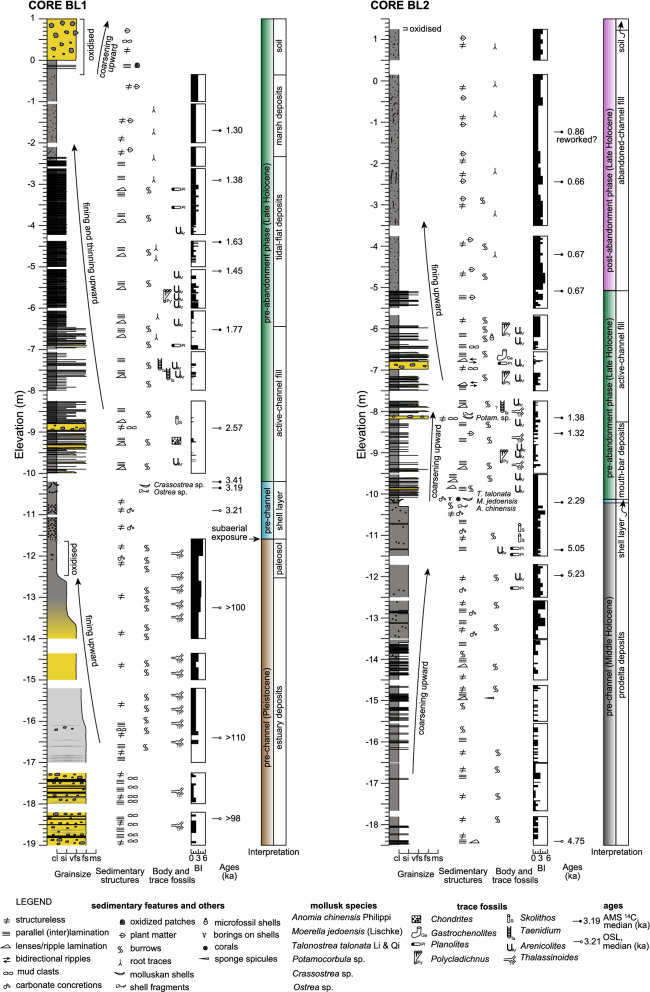
Sedimentological logs of the cores BL1 and BL2 with information about sedimentary features, trace and body fossils, ages and interpretations. Grain-size scale: cl = clay; si = silt; vfs = very-fine sand; fs = fine sand; ms = medium sand.

Nine sedimentary facies (Table 1) were interpreted from the core dataset. Channel deposits overlie shell layers dated at 3.4–3.2 ka and 2.3 ka, which were found in the two longest cores (BL1 and BL2) (Fig. 2); these layers consist of silty clay with abundant shells, suggesting a shelf environment prior to the full development of the Ba Lai channel. Shell layers, in turn, overlie Pleistocene oxidised estuarine deposits (BL1 core) and Mid-Holocene prodelta deposits (BL2 core) (Fig. 2; Supplementary Information 2 and 3); these older deposits represent previous phases of the Mekong system and will not be discussed in detail as these are beyond the scope of this study.

**Table 1:** Characteristics of the sedimentary facies identified in this study.

Phase	Facies	Thickness (m)	Description	Main features	Trace fossils	Body fossils	Sed. acc. rates (cm/yr)	Age range (ka)	Interpretation
Post-abandonment	Soil	0.1 to 1.5	Oxidised structureless mud or sand with root traces	Oxidation, root traces, in some cases coarsening upward	Root traces; BI 4–5				Recent subaerial exposure
	Abandoned-channel fill	1.5 to 6.3	Structureless silty clay with root traces and plant matter	Root traces, plant fragments, burrows, faint parallel lamination	Root traces, unidentified burrows*;* BI 0–5		1.9–11.1	0.73–0.66	Deposition in a channel detached from deltaic network
Pre-abandonment	Marsh deposits	0.5 to 2	Structureless silty clay with root traces and plant matter	Root traces, plant fragments	Root traces, unidentified burrows; BI 1–5		0.8–0.9	1.30	Deposition in upper intertidal to supratidal marsh areas
	Tidal-flat deposits	1 to 4.1	Clay with silt laminae and lenses and root traces	Clay drapes, parallel (inter)lamination, cyclical patterns, lenses, burrows, root traces, fining upward	Root traces, *Planolites*, *Arenicolites, Polykladichnus;* BI 0–5		0.7–1.4	1.77–1.02	Deposition in subtidal to intertidal areas
	Active-channel fill	2.4 to 3.8	Clay with silt to fine sand laminae and lenses	Clay drapes, fluid muds, mud clasts, parallel (inter)lamination, cyclical patterns, (bidirectional) lenses, burrows, fining upward	Root traces, *Arenicolites, Chondrites, Gastrochaenolites, Planolites, Polykladichnus, Skolithos, Taenidium, Thalassinoides;* BI 0–4	*Potamocorbula* sp., microfossils, shell fragments	0.1–0.5	2.57–1.38	Deposition in channel attached to deltaic network
	Mouth-bar deposits	1.7 to > 2.2	Clay to fine sand arranged in (inter)laminae and (inter)bedding with lenses	Clay drapes, fluid muds, mud clasts, parallel (inter)lamination, cyclical patterns, lenses, burrows, coarsening upward	Root traces, *Arenicolites, Thalassinoides, Polykladichnus;* BI 0–3	Shell fragments	0.5–1.4	1.67–1.32	Deposition near river mouth
Pre-channel	Shell layers	0.3 to 1.3	Structureless silty clay with shells or carbonate concretions	Shells, carbonate concretions, borings on shells	*Sedilichnus*	Corals, *Ostrea* Sp., *Crassostrea* sp., *Anomia chinensis* Philippi, *Moerella jedoensis* (Lischke), *Talonostrea talonata* Li & Qi		3.41–2.29	Deposition on shelf, basinward of river mouth
	Mid-Holocene deposits	> 8.3	Alternations of clay and silt or mottled mud	Parallel (inter)lamination, mottling, carbonate concretions, coarsening upward	*Skolithos*, *Planolites*, *Arenicolites*; BI 0–5	Sponge spicules		5.23–4.75	Prodelta
	Pleistocene deposits	> 7.5	Alternations of clay to fine sand, or mottled sand or mud	Clay drapes, fluid muds, mud clasts, parallel (inter)lamination, mottling, oxidation, fining upward	*Thalassinoides;* BI 0–5			> 98	Estuary

**Figure 3:**
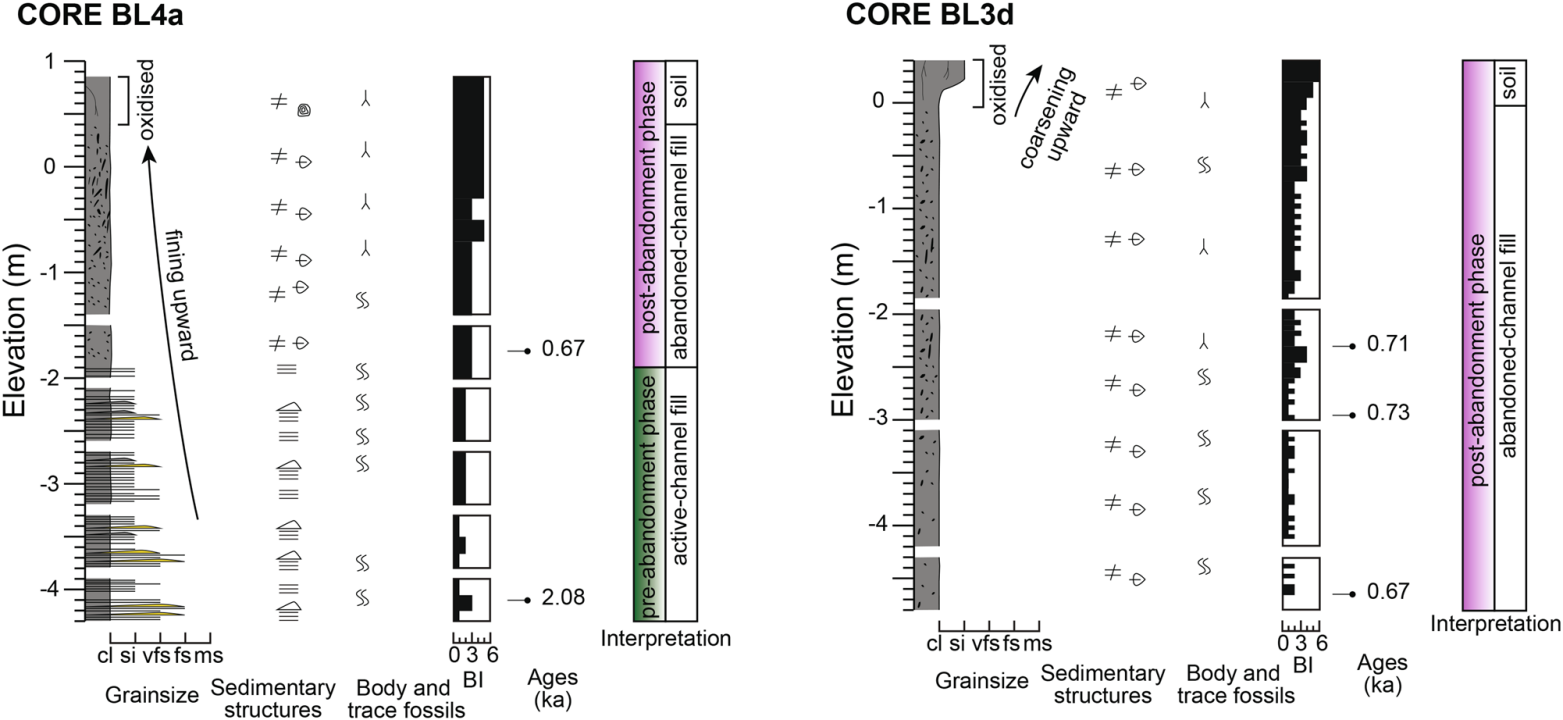
Sedimentological logs of the cores BL4a and BL3d with information about sedimentary features, trace and body fossils, ages and interpretations. Grain-size scale: cl = clay; si = silt; vfs = very-fine sand; fs = fine sand; ms = medium sand. See Fig. 2 for legend.

**Figure 4:**
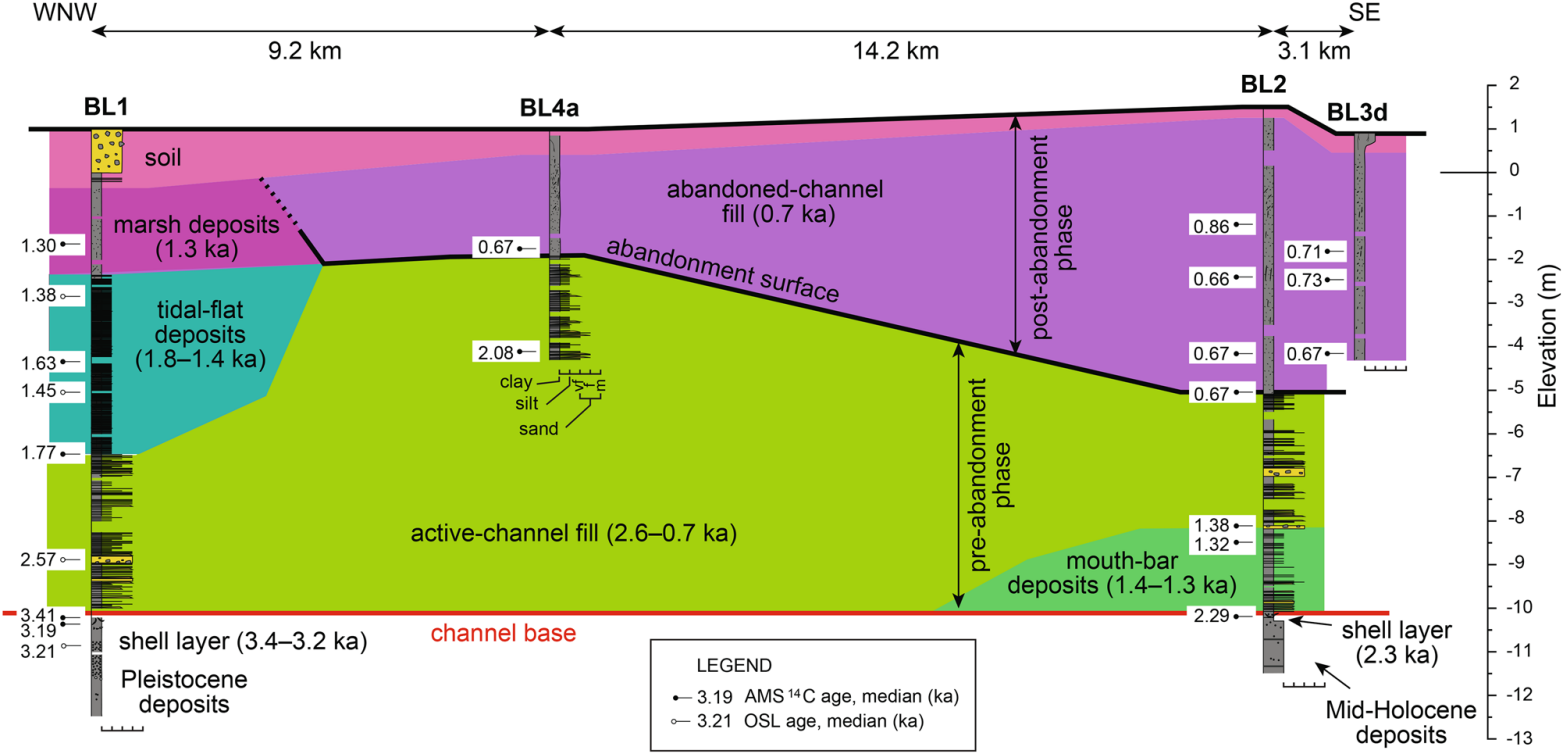
Panel reconstructed based on the four sediment cores showing a longitudinal section of the ancient Ba Lai channel. The section shows the stacking of different sedimentary facies of the pre-abandonment and post-abandonment phases.

**Figure 5:**
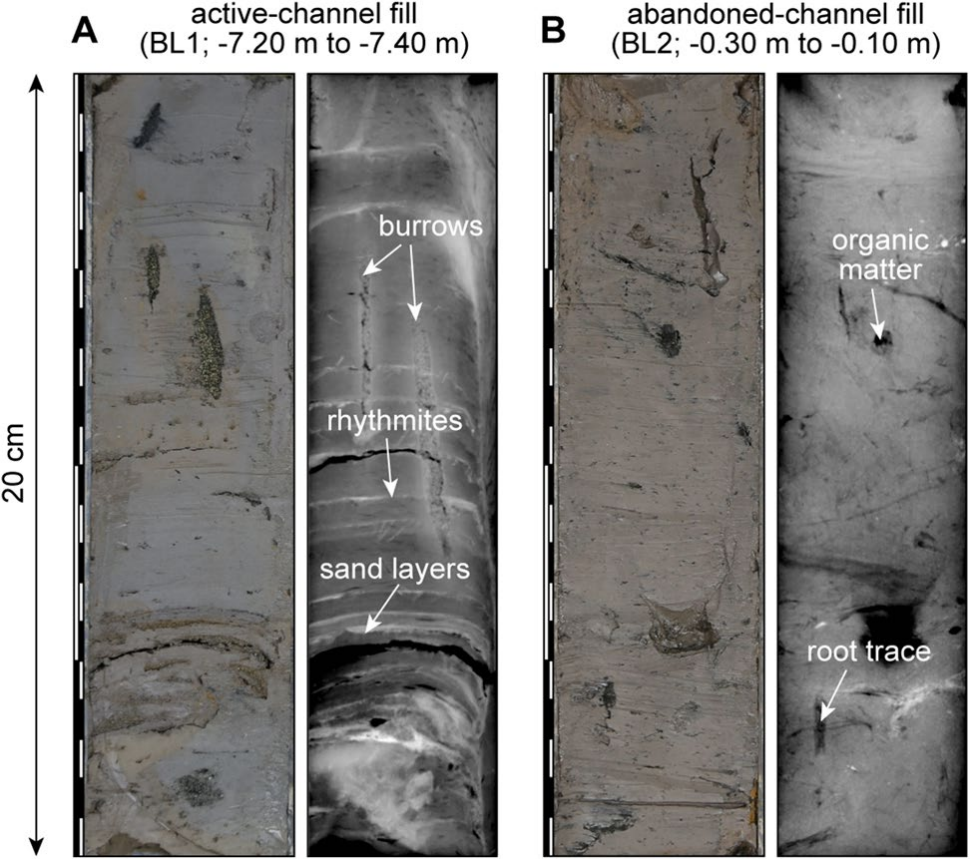
Selected photographs and x-radiographs showing a 20-cm-long part of the cores and highlighting the difference in sedimentary facies between the (**A**) active-channel fill and (**B**) abandoned-channel fill.

The channel base was intersected in two of the cores at elevations of approximately -10 m; considering the elevation of the cored sites, the full thickness of the channel fill is up to 11.6 m. The channel deposits consist of two different phases: (1) pre-abandonment and (2) post-abandonment (Figs. 2 and 3). The pre-abandonment phase includes active-channel fill and mouth-bar deposits (Fig. 4), both consisting of clay with millimetre- to centimetre-scale intercalations of silt to fine-sand laminae and/or lenses with various degrees of bioturbation (Fig. 5A). These two facies are similar but were differentiated based on the morphological evidence of the mouth bar in plan-view (Fig. 1B), higher sediment accumulation rates in mouth-bar deposits and different vertical grain-size trends (fining upward for the active-channel fill and coarsening upward for mouth-bar deposits). The deposits of the pre-abandonment phase are dated between 2.6 ka and 0.7 ka (Fig. 4). In BL1 core, the active-channel phase also comprises tidal-flat and marsh deposits dated between 1.8 ka and 1.3 ka (Fig. 2); they mainly consist of organic-rich structureless or laminated mud, which together with the location of the core (i.e., note the proximity of this core to the palaeochannel margin in Fig. 1B), suggests accumulation in shallow areas near the channel margin.

The post-abandonment phase consists of the abandoned-channel fill, with a maximum thickness of 6.4 m (BL2 core), capped by soil consisting of oxidised sand and/or mud with root traces. The contact with the underlying pre-abandonment deposits, observed in cores BL2 and BL4a, is sharp and characterised by an abrupt disappearance of the sand fraction and an increase in organic matter. The abandoned-channel fill consists of organic-rich silty clay with abundant plant fragments and root traces (Fig. 5B); this facies is structureless or shows a faint lamination with millimetre-scale layers of plant fragments. The abandoned-channel fill shows similar features to marsh deposits, but the two facies were differentiated based on the stratigraphic context, different ages and higher thicknesses and sediment accumulation rates in the abandoned-channel fill compared to marsh deposits. Seven of the eight ages from the abandoned-channel fill indicate consistent values at 0.7 ka (Fig. 4), whereas the remaining value is 0.9 ka, but defining an age reversal (i.e., an older age above younger ages).

## Evolution of the Ba Lai palaeochannel

**Figure 6:**
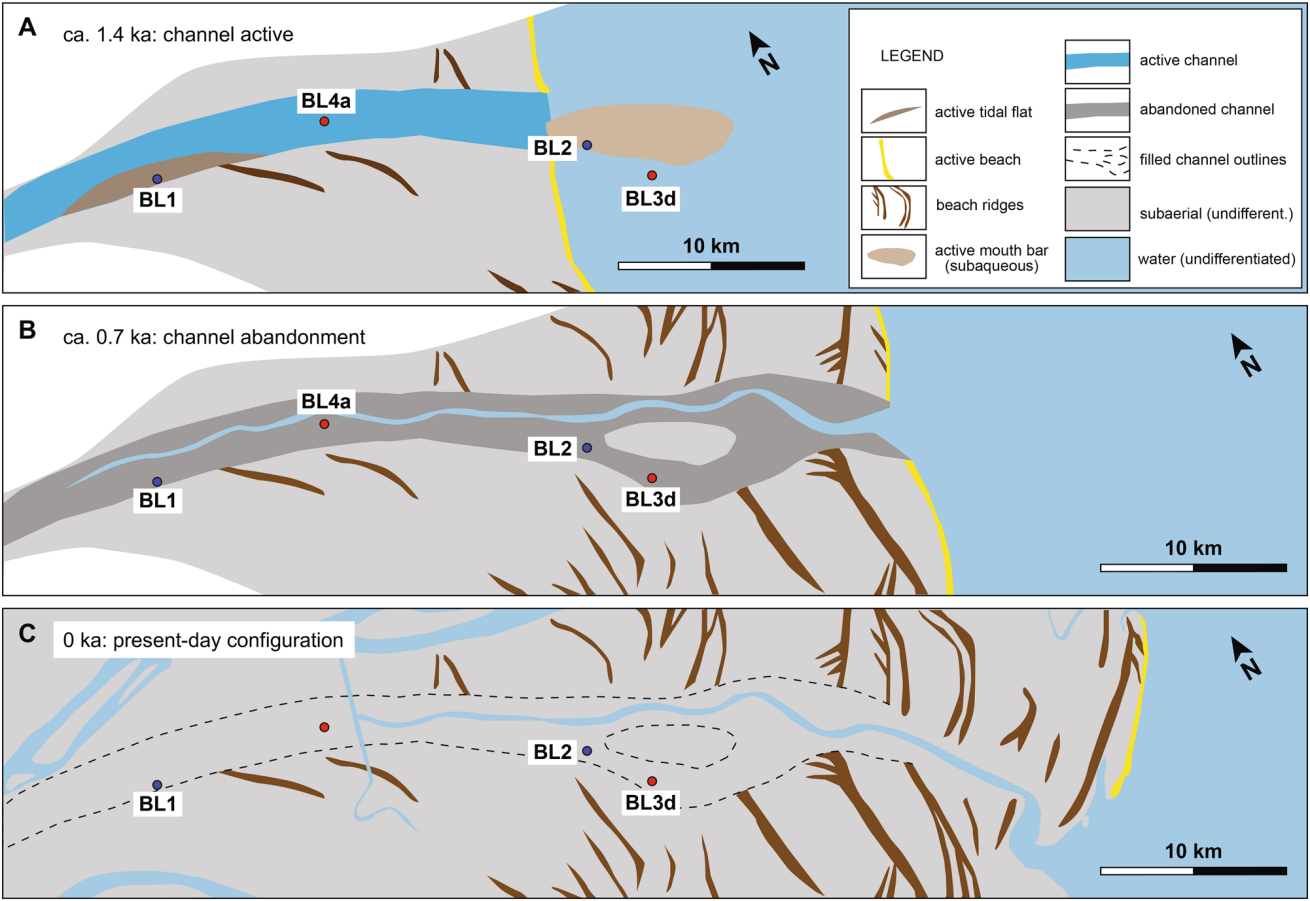
Palaeogeographic reconstructions showing (**A**) the active phase of the Ba Lai channel, (**B**) its abandonment, and (**C**) the present-day configuration. White parts indicate areas of uncertainty regarding the evolution of the neighbouring channels. Beach ridges were identified using the DEM and^21^. The palaeogeographies also show the progradation of the shoreline changing from symmetrical to asymmetrical when the channel was abandoned.

The present dataset constrains the evolution of the ancient Ba Lai palaeochannel of the MRD. The channel was active and connected to the deltaic network between 2.6 ka and 0.7 ka (Fig. 6A). The deposits of this phase consist of a mixture of sand and mud and are similar to those of the present-day active distributary channels and river-mouth areas of the MRD^14^, implying similar hydro-sedimentary dynamics; this similarity and the context of the Ba Lai palaeochannel (i.e., located within the tide-dominated part of the MRD) both suggest that this was a tide-dominated distributary channel originally attached to the deltaic network and with acting riverine and tidal processes. It is suggested that the high amounts of mud in the active-channel fill are due to the tidal dynamics and the formation of a turbidity maximum in the channel, whereas the presence of sand suggests a relatively strong river input. During this phase, the channel was building a subaqueous mouth bar, whose outlines are still partially visible today (Fig. 1B). It is suggested that this bar gradually became subaerial and was incorporated within the channel due to the progradation; its preservation was possible because the bar was an elevated feature and/or subaerial and was only partially buried by mud when the channel was abandoned. Although the channel was still active, it should be noted that some areas near the channel margins (e.g., near the BL1 core) had already accumulated enough sediment to transition into tidal flats or subaerial marshes before the channel abandonment; this is indicated by the older ages of these deposits compared to the abandoned-channel fill (Fig. 4).

At 0.7 ka, the channel was abruptly abandoned and rapidly infilled with sediment (Fig. 6B), as suggested by the nearly coeval ages from the abandoned-channel fill (Fig. 4). The abandoned-channel fill mainly consists of organic-rich structureless mud and is similar to the facies of other abandoned channels reported from modern and ancient systems [e.g., ^4,19,20^]. Subsequent to the detachment of the Ba Lai channel from the deltaic network, tidal currents were likely the main acting process and were rapidly infilling the channel importing sediment from the mouth. Considering the longshore sediment transport toward the southwest characterizing the MRD, it is suggested that this sediment was delivered to the shelf by distributary channels in the north-eastern part of the MRD (e.g., the My Tho channel; Fig. 1B), successively resuspended, transported toward the Ba Lai channel mouth and eventually imported. During this phase, river currents in the abandoned Ba Lai channel were either absent or strongly reduced to the point of being able to supply directly to the channel only the mud fraction.

Finally, the infilled channel became part of the subaerial delta plain as it is observed in the present-day configuration (Fig. 6C). The recent progradation of the deltaic shoreline near the Ba Lai palaeochannel is also deducted by the presence of mouth-bar deposits and beach ridges. The progradation rate of this shoreline was estimated based on the chronology revealed by OSL ages of beach ridges from^21^. When the channel was active, progradation with average rates of 34–45 m/yr was consistent across the shoreline. After channel abandonment progradation still occurred, but it was highly asymmetrical; 31 m/yr north of the channel and 7 m/yr south of the channel. Asymmetry in progradation was because the progradation of this portion of the shoreline was not anymore sustained by the Ba Lai channel but from the channels located in the north-eastern part of the MRD^21^; this implies that the longshore transport from northeast to southwest that today characterizes the MRD has also been active in the last 0.7 kyr.

## Sediment volumes, accumulation rates and timing of infilling

**Figure 7:**
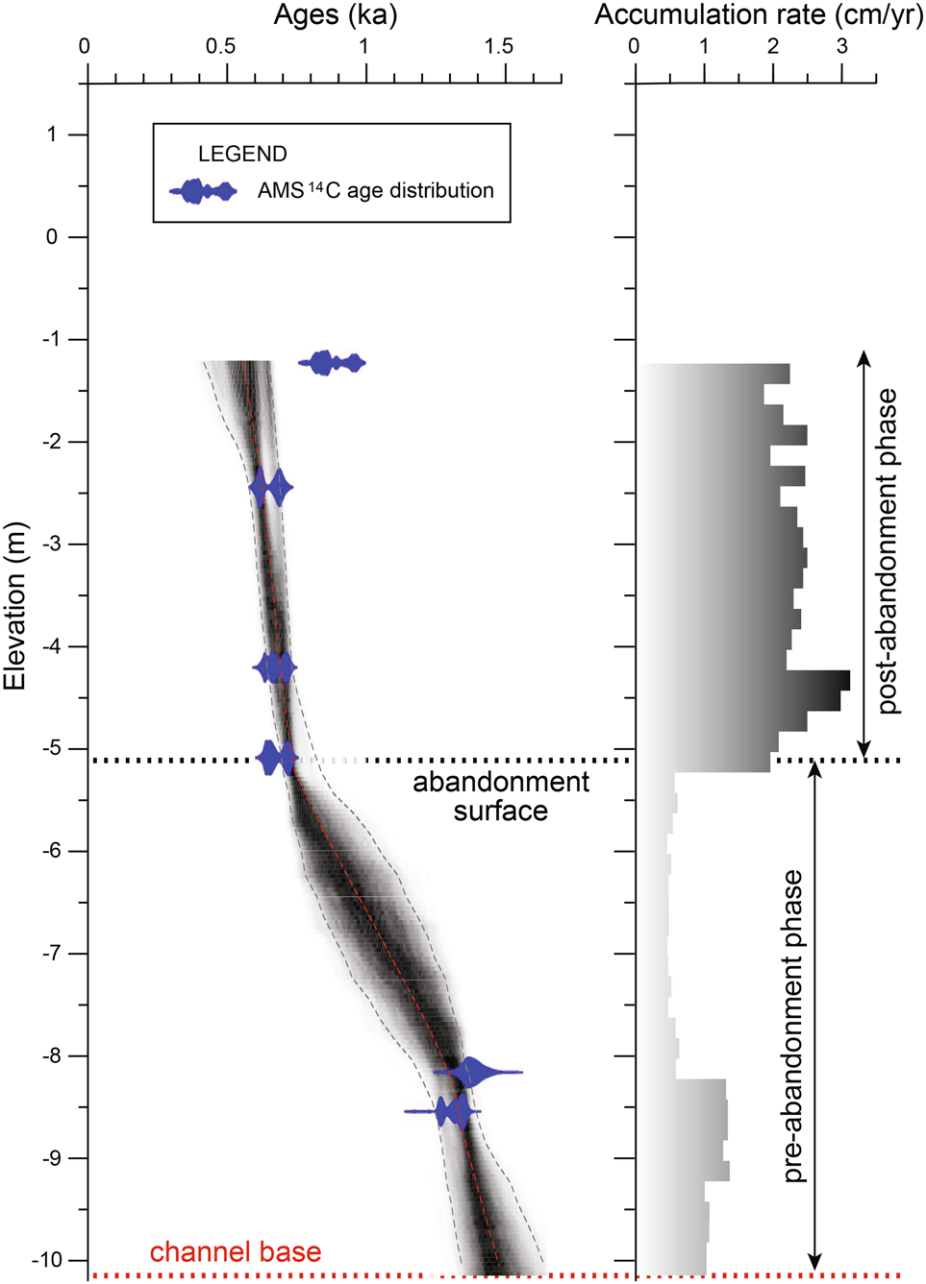
Age-depth model and sediment accumulation rates of the core BL2 based on the AMS ^14^C ages.

The palaeochannel is approximately 11 m thick, 3 km wide, and 50 km long, corresponding to an estimated volume of 1.3 km^3^, which is roughly equally subdivided between the pre-abandonment and post-abandonment deposits. If considering an average thickness of 5 m along the central part, the volume of the abandoned-channel fill is estimated to be 0.6 km^3^; this corresponds, using a dry bulk density of 1 g/cm^3^^22^, to approximately 600 Mt of sediment that was rapidly stored in the abandoned channel after its detachment. This sediment stored as abandoned-channel-fill consists of organic-rich mud, implying that similar abandoned channels can sequester a relatively important amount of organic carbon that should be taken into account when evaluating local and global carbon budgets [e.g., ^23–25^]. In the BL2 core, sediment accumulation rates of the active-channel fill are constantly 0.5 cm/yr, although they are slightly higher in the associated mouth-bar deposits (Fig. 7). Sediment accumulation rates increase abruptly in correspondence with the abandonment surface, with the abandoned-channel fill in core BL2 having values ranging from 1.9 cm/yr to 3.1 cm/yr (Fig. 7). Similar values are also observed in the other cores, with the active-channel fill having sedimentation rates of 0.1–0.5 cm/yr in the cores BL1 and BL4a, and the abandoned-channel fill even reaching 11.1 cm/yr in the BL3d core. Therefore, during its active phase the channel mainly aggraded at a few millimetres per year, which is consistent, for example, with active channels in anastomosed fluvial systems^4^. By contrast, during the abandoned phase the channel aggraded much faster, in the order of centimetres to decimetres per year.

**Figure 8:**
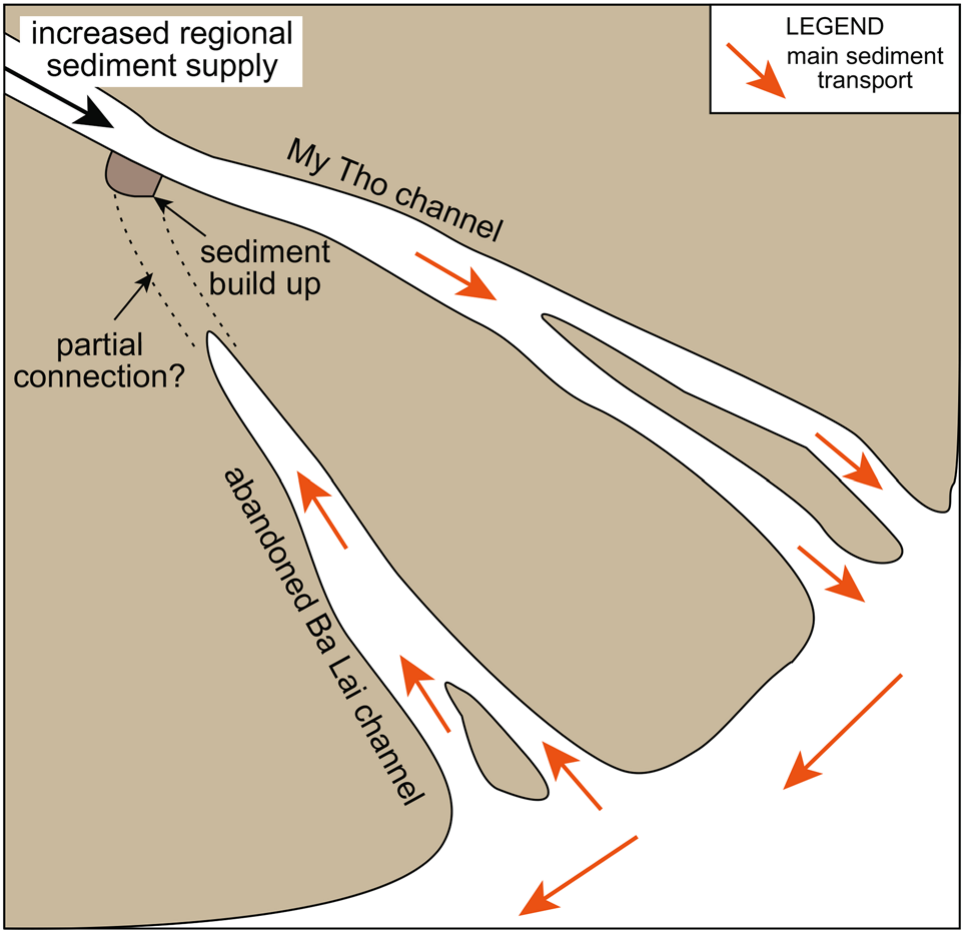
Schematic model showing the abandonment of the Ba Lai channel due to a sediment buildup and the infill with sediment from its mouth.

The consistent ages at 0.7 ka for the abandoned-channel fill suggest that the channel was filled rapidly in the order of decades or maximum a few centuries. Using the 2-sigma distribution of AMS ^14^C ages from the abandoned-channel fill and excluding one reversed age (Supplementary Information 2), the difference between the absolute minimum and maximum ages is 232 years; this value might suggest an approximate time range in which the abandoned channel was infilled. Also, given an average thickness of 5 m and sediment accumulation rates between 1.9 cm/yr and 11.1 cm/yr for the abandoned-channel fill, it is calculated that the channel could have been infilled in as little as 45–263 years after its abandonment. Furthermore, an additional consideration can be made based on the MRD sediment supply. At quasi-natural conditions (i.e., before dams, sand mining, etc.), the sediment discharge of the MRD was estimated in 160 Mt/yr^26^, of which approximately 27% flows through the My Tho channel located north-eastward of the Ba Lai^14^. If assuming that the delta configuration and discharge were similar at 0.7 ka, it could be expected that the sediment exported by the My Tho channel would be transported predominantly south-westward due to the dominant current in the area; part of this sediment would have been reimported to fill the Ba Lai channel (Fig. 8). If assuming that one fifth of the sediment annually exported by the My Tho would have been used to infill the abandoned channel, the complete fill of the latter could have occurred in 69 yr. If assuming one tenth of the sediment, the fill could have been completed in 139 yr. These values seem realistic considered the dynamics of the MRD and are included within the time range calculated based on the ages and on the thickness and sediment accumulation rates of the abandoned-channel fill, suggesting that this time-range estimation could be considered reliable.

## Mechanisms for abandonment and infill of tide-dominated deltaic channels

Due to the uniqueness of this case study, issues arise when comparing the evolution and abandonment mechanisms of the Ba Lai palaeochannel with other known examples. The abandonment recorded in the deposits of the Ba Lai palaeochannel was abrupt and the infilling with sediment was rapid. This is similar to what is observed in oxbow-lake fills in fluvial environments^19^, but nonetheless, the plan-view straight morphology of the Ba Lai palaeochannel clearly contrasts with meander cutoffs, excluding this as a potential mechanism. Also, it should be noted that large-scale and frequent avulsions, as seen in the Yellow and Mississippi rivers^27,28^ as well as other river-dominated systems, are rare or absent in large-scale tide-dominated deltas, including the Mekong^8^. For example, the fluvial-dominated Yellow River Delta in China experienced seven major avulsions in the last 0.2 kyr^27^, which occurred with abrupt relocations of the river channel following a breach in the levee during floods^29^. The number of avulsions in fluvial systems is even higher, in the order of tens within a century, if considering smaller-scale adjustments like the ones reported from the Rio Grande in Colorado^2^. In the MRD, floods occur yearly, but only one major abandoned channel exists, suggesting that an avulsion node is not present. We suggest that in the case of the Ba Lai channel the abandonment and rapid infill occurred because its upstream entrance at the bifurcation point was blocked by a buildup of sediments (Fig. 8). Similar sediment buildups are reported also from fluvial systems and associated to floods and/or channel margin failures^3,30^, although the abandonment mechanisms for the Ba Lai remain fundamentally different. The length of the Ba Lai channel is approximately 50 km and roughly coincides with the length of maximum saltwater intrusion in the MRD^14^, suggesting that the combined river-tidal dynamics that control the saltwater intrusion might have also played a role in causing the sediment buildup and abandonment of the Ba Lai channel. This suggests that the abandonment of the Ba Lai is likely linked to autogenic mechanisms of tide-dominated deltaic systems, although these are not fully understood yet. A current abandonment of a tide-dominated distributary channel in the North Branch of the Yangtze River Delta, eastern China, is only partially analogue to this study; this channel has naturally and gradually been evolving from an active distributary to an estuary since 0.2 ka, but it has not been entirely filled despite a high sedimentation rate^31–33^. The rapid infill and uniqueness of the Ba Lai palaeochannel in the MRD suggest that its abandonment was also favoured by an exceptional event possibly linked to allogenic factors. In fact, this abandonment occurred synchronously with a long-term increase in sediment supply documented at Camau, in the southwestern part of the MRD^34^. According to^34^, the increase in sediment supply was driven by a change in land use in the upstream basin due to the establishment of the Min Dynasty in the fourteenth century and/or by a change in regional climate with intensification of the winter monsoon that drives the longshore transport. This study highlights that mechanisms for abandonment and infilling of tide-dominated deltaic channels do not entirely fit widely used models developed for fluvial-dominated environments. Abandoned tide-dominated deltaic channels are rare in nature and their mechanisms are not fully understood yet. Their abandonment might be driven by autogenic factors related to the river-tidal and deltaic dynamics and, in some cases, favoured by allogenic factors, such as human impact and/or climate change.

## Methods

Four sediment cores (BL1, BL2, BL3d and BL4a) were collected from the ancient Ba Lai channel in 2019 (Supplementary Information 1: core dataset). The margins of the palaeochannel were constrained in QGIS using a Digital Elevation Model (DEM) from the Shuttle Radar Topography Mission (SRTM) obtained from the United States Geological Survey (https://earthexplorer.usgs.gov/) and satellite images from the Google Plugin. The surface elevations of the cores were estimated from topographic maps. In the laboratory, cores were split, photographed, described and sampled for x-radiography, age dating and shell identification. The description included the recording of the sediment grain size, vertical trends, sedimentary structures and trace and body fossils, which were used to differentiate nine sedimentary facies (Table 1). Bioturbation diversity and intensity were also recorded using the bioturbation index (BI) of^35^. X-radiographs were acquired for selected intervals using 20-cm-long slabs with a SOFRON SRO-i503-2 (Sofron Inc., Tokyo, Japan) instrument coupled with a digital X-ray detector NAOMI NX-04SN (RF Inc., Nagano, Japan). Mollusc species from four samples were photographed, identified and interpreted in terms of depositional environments based on ecological data of^36^. Accelerator Mass Spectrometry radiocarbon (AMS ^14^C) dating was carried out at Beta Analytic Inc. on samples of plant matter and shells. The conventional ages were calibrated using the software Calib version 7.1.0 applying the curves IntCal13 for plant material samples and MARINE13 for shell samples^37–39^. A delta R of -60 years, which was a weighted mean of two data points near the MRD region^40,41^, was used for shells samples. Optical-Stimulated-Luminescence (OSL) dating of fine-grained quartz was conducted at the Geological Survey of Japan (Supplementary Information 3: OSL ages) following the procedure outlined in^42^. In this paper, both AMS^14^C and OSL ages are expressed in ka relative to 2020 CE. Age-depth models and sediment accumulation rates were calculated using the Bacon age-modelling software^43^, with depth data every 10–20 cm and ages calculated to the depth of the uppermost dating sample. All figures were prepared with the software Adobe Illustrator 2020 version 24.3.

## Supplementary Information

Supplementary file 1.

Supplementary file 2.

Supplementary file 3.

## Data Availability

All data are either published within this manuscript and its supplementary information or available on requests.
