# Supplementary material for: Abandonment and rapid infilling of a tide-dominated distributary channel at 0.7 ka in the Mekong River Delta
Source: Sci Rep. 2021 May 26;11:11040. doi: 10.1038/s41598-021-90268-6 (PMC8154897; doi:10.1038/s41598-021-90268-6)
Supplement: Supplementary file 1 [file 41598_2021_90268_MOESM1_ESM.docx]

**Abandonment and Rapid Infilling of a Tide-Dominated Distributary Channel at 0.7 ka in the Mekong River Delta**

Marcello Gugliotta, Yoshiki Saito, Thi Kim Oanh Ta, Van Lap Nguyen, Toru Tamura, Zhanghua Wang, Andrew D. La Croix, Rei Nakashima

Supplementary Information 1. Details of the core-based dataset used in this study.

| Core | Site latitude (WGS84) | Site longitude (WGS84) | Surface elevation  (m) | Core type | Core length (m) | Core recovery (%) |
| --- | --- | --- | --- | --- | --- | --- |
| BL1 | 10.286107 | 106.340354 | 1.0 | borehole | 20.0 | 89.7 |
| BL2 | 10.195000 | 106.527151 | 1.5 | borehole | 20.0 | 89.8 |
| BL3d | 10.171614 | 106.542891 | 0.9 | peat corer | 5.2 | 90.4 |
| BL4a | 10.269657 | 106.423911 | 1.0 | peat corer | 5.3 | 86.8 |
