# Supplementary material for: Abandonment and rapid infilling of a tide-dominated distributary channel at 0.7 ka in the Mekong River Delta
Source: Sci Rep. 2021 May 26;11:11040. doi: 10.1038/s41598-021-90268-6 (PMC8154897; doi:10.1038/s41598-021-90268-6)
Supplement: Supplementary file 2 [file 41598_2021_90268_MOESM2_ESM.docx]

**Abandonment and Rapid Infilling of a Tide-Dominated Distributary Channel at 0.7 ka in the Mekong River Delta**

Marcello Gugliotta, Yoshiki Saito, Thi Kim Oanh Ta, Van Lap Nguyen, Toru Tamura, Zhanghua Wang, Andrew D. La Croix, Rei Nakashima

Supplementary Information 2. Details of the AMS ^14^C dating.

| **Core** | **Sample elevation (m)** | **Material** | **Conventional age**  **(yr BP)** | **Calibrated age median (cal BP)** | **2 sigma range (cal BP)** | **Probability (cal BP)** | **δ^13^C (‰)** |
| --- | --- | --- | --- | --- | --- | --- | --- |
| BL1 | -1.70 | plant | 1270 ± 30 | 1225 | 1287–1173 | 0.968 | -28.99 |
|  |  |  |  |  | 1158–1147 | 0.013 |  |
|  |  |  |  |  | 1131–1129 | 0.002 |  |
|  |  |  |  |  | 1108–1191 | 0.017 |  |
| BL1 | -4.40 | plant | 1660 ± 30 | 1563 | 1689–1671 | 0.030 | -24.49 |
|  |  |  |  |  | 1624–1511 | 0.939 |  |
|  |  |  |  |  | 1457–1440 | 0.021 |  |
|  |  |  |  |  | 1432–1422 | 0.010 |  |
| BL1 | -6.52 | plant | 1780 ± 30 | 1699 | 1812–1748 | 0.182 | -27.54 |
|  |  |  |  |  | 1745–1616 | 0.818 |  |
| BL1 | -10.20 | shell | 3400 ± 30 | 3341 | 3426–3242 | 1.000 | -4.02 |
| BL1 | -10.35 | shell | 3230 ± 30 | 3124 | 3226–3010 | 1.000 | -3.95 |
| BL2 | -1.23 | plant | 880 ± 30 | 788 | 908–845 | 0.281 | -27.99 |
|  |  |  |  |  | 833–729 | 0.719 |  |
| BL2 | -2.44 | plant | 560 ± 30 | 593 | 642–588 | 0.523 | -28.53 |
|  |  |  |  |  | 564–522 | 0.477 |  |
| BL2 | -4.20 | plant | 620 ± 30 | 601 | 658–551 | 1.000 | -29.65 |
| BL2 | -5.08 | plant | 640 ± 30 | 599 | 667–621 | 0.426 | -29.85 |
|  |  |  |  |  | 610–554 | 0.574 |  |
| BL2 | -8.15 | shell | 1700 ± 30 | 1307 | 1374–1251 | 1.000 | -5.53 |
| BL2 | -8.53 | plant | 1310 ± 30 | 1253 | 1294–1224 | 0.719 | -26.84 |
|  |  |  |  |  | 1212–1182 | 0.281 |  |
| BL2 | -10.20 | shell | 2480 ± 30 | 2217 | 2303–2123 | 1.000 | -0.80 |
| BL2 | -11.35 | plant | 4410 ± 30 | 4981 | 5257–5249 | 0.006 | -27.61 |
|  |  |  |  |  | 5231–5226 | 0.003 |  |
|  |  |  |  |  | 5214–5189 | 0.041 |  |
|  |  |  |  |  | 5054–4866 | 0.950 |  |
| BL2 | -11.98 | plant | 4520 ± 30 | 5158 | 5305–5212 | 0.337 | -28.60 |
|  |  |  |  |  | 5193–5050 | 0.663 |  |
| BL3d | -1.80 m | plant | 670 ± 30 | 641 | 676–631 | 0.559 | -31.80 |
|  |  |  |  |  | 599–559 | 0.441 |  |
| BL3d | -2.45 m | plant | 700 ± 30 | 664 | 688–642 | 0.820 | -28.10 |
|  |  |  |  |  | 589–564 | 0.180 |  |
| BL3d | -4.15 m | plant | 640 ± 30 | 599 | 667–621 | 0.426 | -26.89 |
|  |  |  |  |  | 610–554 | 0.574 |  |
| BL4a | -1.73 m | plant | 630 ± 30 | 600 | 663–618 | 0.408 | -28.37 |
|  |  |  |  |  | 613–552 | 0.592 |  |
| BL4a | -4.10 m | plant | 2050 ± 30 | 2013 | 2113–2076 | 0.124 | -27.45 |
|  |  |  |  |  | 2072–1932 | 0.876 |  |
