# Supplementary material for: Abandonment and rapid infilling of a tide-dominated distributary channel at 0.7 ka in the Mekong River Delta
Source: Sci Rep. 2021 May 26;11:11040. doi: 10.1038/s41598-021-90268-6 (PMC8154897; doi:10.1038/s41598-021-90268-6)
Supplement: Supplementary file 3 [file 41598_2021_90268_MOESM3_ESM.docx]

**Abandonment and Rapid Infilling of a Tide-Dominated Distributary Channel at 0.7 ka in the Mekong River Delta**

Marcello Gugliotta, Yoshiki Saito, Thi Kim Oanh Ta, Van Lap Nguyen, Toru Tamura, Zhanghua Wang, Andrew D. La Croix, Rei Nakashima

Supplementary Information 3. Details of the OSL dating. D_e_ = equivalent dose. Numbers of aliquots measured are shown with those used for the determination of *D_e_* in parenthesis. *D_e_* of the samples at -13.25 m, -16.40 m and -18.36 m in BL1 was above the saturation level, and 2*D_0_* is shown here instead and used for estimating a minimum age.

| **Core** | **Sample elevation (m)** | **Material** | **Grain size (μm)** | **K (%)** | **U (ppm)** | **Th (ppm)** | **Rb (ppm)** | **Water content (%)** | **Dose rate (Gy)** | **D_e_ (Gy)** | **Age (ka)** | **No. aliquots** |
| --- | --- | --- | --- | --- | --- | --- | --- | --- | --- | --- | --- | --- |
| BL1 | -2.90 | quartz | 4–11 | 1.95 | 2.83 | 14.2 | 138 | 63 | 2.63 ±0.14 | 3.62 ±0.07 | 1.38 ±0.08 | 6 (6) |
| BL1 | -5.10 | quartz | 4–11 | 1.86 | 2.65 | 13.8 | 137 | 60 | 2.54 ±0.13 | 3.68 ±0.05 | 1.45 ±0.08 | 6 (5) |
| BL1 | -8.90 | quartz | 4–11 | 1.59 | 2.66 | 19.1 | 110 | 45 | 2.95 ±0.17 | 7.57 ±0.10 | 2.57 ±0.15 | 6 (5) |
| BL1 | -10.90 | quartz | 4–11 | 1.94 | 3.26 | 15.6 | 144 | 71 | 2.58 ±0.13 | 8.27 ±0.11 | 3.21 ±0.17 | 6 (5) |
| BL1 | -13.25 | quartz | 120–180 | 0.79 | 1.41 | 11.1 | 63.2 | 21 | 1.57 ±0.12 | > 162 | > 100 | 6 (6) |
| BL1 | -16.40 | quartz | 120–180 | 1.23 | 1.11 | 7.45 | 71.3 | 18 | 1.69 ±0.13 | > 182 | > 110 | 6 (3) |
| BL1 | -18.36 | quartz | 120–180 | 1.43 | 1.25 | 8.11 | 88.1 | 20 | 1.88 ±0.15 | > 185 | > 98 | 6 (5) |
| BL2 | -18.40 | quartz | 4–11 | 1.76 | 2.60 | 13.1 | 118 | 46 | 2.62 ±0.15 | 12.47 ±0.17 | 4.75 ±0.28 | 6 (5) |
